# Uncovering Druggable Targets in Aortic Dissection: An Association Study Integrating Mendelian Randomization, pQTL, and Protein–Protein Interaction Network

**DOI:** 10.3390/biomedicines12061204

**Published:** 2024-05-29

**Authors:** Daisong Jiang, Sihao Zheng, Xiaokang Xu, Honghua Yue, Weitao Liang, Zhong Wu

**Affiliations:** Department of Cardiovascular Surgery, West China Hospital, Sichuan University, No. 37 Guoxue Road, Wuhou District, Chengdu 610041, China; daisongjiang1998@163.com (D.J.);

**Keywords:** GWAS, mendelian randomization, aortic dissection, therapeutic targets, druggable proteins

## Abstract

Aortic dissection (AD) is a life-threatening acute aortic syndrome. There are limitations and challenges in the discovery and application of biomarkers and drug targets for AD. Mendelian randomization (MR) analysis is a reliable analytical method to identify effective therapeutic targets. We aimed to identify novel therapeutic targets for AD and investigate their potential side-effects based on MR analysis. Data from protein quantitative trait loci (pQTLs) were used for MR analyses to identify potential therapeutic targets. We probed druggable proteins involved in the pathogenesis of aortic dissection from deCODE. In this study, a two-sample MR analysis was conducted, with druggable proteins as the exposure factor and data on genome-wide association studies (GWAS) of AD as the outcome. After conducting a two-sample MR, summary data-based Mendelian randomization (SMR) analysis and colocalization analysis were performed. A protein–protein interaction (PPI) network was also constructed to delve into the interactions between identified proteins. After MR analysis and the Steiger test, we identified five proteins as potential therapeutic targets for AD. SMR analysis and colocalization analysis also confirmed our findings. Finally, we identified ASPN (OR = 1.36, 95% CI: 1.20, 1.54, *p* = 4.22 × 10^−5^) and SPOCK2 (OR = 0.57, 95% CI: 0.41, 0.78, *p* = 4.52 × 10^−4^) as the core therapeutic targets. Through PPI network analysis, we identified six druggable targets, enabling the subsequent identification of six promising drugs from DrugBank for treating AD. This discovery of specific proteins as novel therapeutic targets represents a significant advancement in AD treatment. These findings provide more effective treatment options for AD.

## 1. Introduction

Aortic dissection (AD) is a life-threatening and acute aortic syndrome characterized by an intimal tear that results in the separation of the aortic lumen into true and false lumens [1]. Worldwide, the incidence of AD is estimated to range from five to thirty cases per million people annually, equal to approximately one case per 10,000 individuals per year. Studies have indicated a 52% increase in the incidence of AD among males and a 28% increase in the incidence of AD among females over the past four decades. The mortality rate of untreated ascending AD increases by approximately 1% to 2% per hour within the first 24 to 48 h, reaching up to 50% within the first two days after the incidence of AD. Mortality rates remain significantly high even among hospitalized patients with acute AD [2].

Due to the significant lethality associated with acute aortic dissection, especially Stanford type A dissection that involves the ascending aorta, it needs immediate treatment irrespective of how far the dissection extends distally. The survival of patients with AD relies on the timing of treatment and the success of surgical interventions [3].

The exact causes of AD are not fully understood, but hypertension, arteriosclerosis, vascular wall abnormalities, and genetic factors may be involved. Although we have discovered more genetic information and signaling pathways associated with AD, antihypertensive medications and aortic replacement surgery remain the most important treatment options. Current pharmacological treatments for aortic dissection include beta-blockers, angiotensin-converting enzyme inhibitors, and calcium-channel blockers [4]. More pharmacological interventions or preventative treatments are needed for AD [5].

Genetic variants that affect RNA and protein levels are known as expression quantitative trait loci (eQTLs) and protein quantitative trait loci (pQTLs), respectively. Variations in these specific genetic regions, particularly pQTLs, are closely linked to the alterations in protein expression levels. Analyzing these genetic variants can uncover how they can modify the physical attributes of an organism by influencing the quantity of protein production. eQTL analysis focuses on mRNA levels, while pQTL analysis concentrates on tracking differences in protein levels. pQTLs can help fundamentally understand the implications of certain genetic variations in human diseases [6]. Identifying pQTLs linked to crucial biomarkers or disease-related characteristics is critical for drug discovery, mechanistic studies, and the development of tailored therapeutic strategies [7]. Recognizing and leveraging these genetic insights pave the way for more targeted and personalized interventions [8,9].

Mendelian randomization (MR) is a genetic epidemiology approach that utilizes genetic variants, known as instrumental variables, to explore the causal relationships between modifiable risk factors and health outcomes. These genetic variants are typically single-nucleotide polymorphisms (SNPs) that are associated with the exposure of interest and are used to mimic the effects of randomized controlled trials. The correct application of MR relies on several key assumptions, including the relevance, independence, and exclusion restriction criteria.

In this analytical framework, we adopted a series of parameterized statistical methods, such as the Wald ratio, inverse variance weighted (IVW) method, tests for heterogeneity and pleiotropy, and the Steiger directionality test. Due to their random allocation, MR utilizes genetic variations as instrumental variables for the relationship between exposures and outcomes, which minimizes the effect of confounding factors, offering a more stringent method for investigating causal relationships. This makes MR a powerful tool for assessing potential causal relationships in genetic data, providing a scientific basis for disease prevention and development of new treatments.

Human proteins play pivotal roles in various biological processes and are primary drug targets. Previous MR studies using pQTL have shown a potential causal relationship between AD and specific circulating proteins, such as cathepsin B and proteins involved in the renin–angiotensin system (Ras) signaling pathway [10,11]. Although these findings reveal the potential of certain circulating proteins as biomarkers for AD, the current treatments for AD remain insufficient. There is an urgent need for further evidence on targeted treatment for AD.

Genome-wide association studies (GWAS) can help identify the associations between genotypes and phenotypes by detecting differences in allele frequencies of genetic variations. GWAS employs high-throughput genomic sequencing technologies to analyze genetic markers, such as single-nucleotide polymorphisms (SNPs), to pinpoint genetic variations associated with specific diseases or phenotypes (observable traits) [12,13].

MR analyses use genetic instrumental variables, typically SNPs from GWAS, to estimate the causal effect of an exposure on the outcome [14,15]. The principle of drug-targeted MR involves leveraging genetic variants as instruments to modulate the causal effects of specific genes or proteins on specific outcomes. This approach aims to identify potential pharmacological interventions that can repair or regulate these biological targets and improve patients’ prognosis. Pharmacological interventions identified by this method may repair the damaged vascular wall, modulate endothelial cell function to enhance vascular integrity, or attenuate the inflammatory response leading to AD. Such drugs might exert their therapeutic effects by influencing specific signaling pathways or molecular mechanisms. A deeper mechanistic insight, obtained from targeted research and clinical trials, is crucial to elucidate how interventions identified through drug-targeted MR can effectively treat AD. A few studies have integrated GWAS and pQTL data to investigate AD within the framework of MR. MR-based strategies have shown promise in identifying potential therapeutic targets across a range of diseases, underscoring their potential in uncovering novel interventions for AD.

The current studies highlight the potential of MR to elucidate the causal relationship between genetic predisposition and disease outcome. By leveraging this approach, researchers can identify new pathways for developing targeted treatments and preventive measures for complex diseases, such as AD. The ability of MR to identify genetic determinants offers a foundation for developing innovative therapeutic strategies. MR analysis can identify genetic variants associated with drug metabolism, efficacy, and side-effects in AD. These findings support the findings of preclinical studies.

## 2. Materials and Methods

### 2.1. Selection of Druggable Genes

Ensembl is a bioinformatics project designed to provide a set of automated genomic annotations, jointly developed and maintained by the European Bioinformatics Institute (EBI) in the United Kingdom and the Wellcome Trust Sanger Institute in Hinxton. In Ensembl v.73, 4479 of the annotated protein-coding genes are considered to be druggable or potentially druggable and are divided into three tiers [16] (Table S1). The first group of 1427 genes included the efficacy targets of approved small-molecule and biological therapeutic drugs and clinical-stage drug candidates. The second group included 682 genes encoding targets with known bioactive, drug-like, small-molecule binding partners and ≥50% identity with approved drug targets. The third class of 2370 genes encoded secretory or extracellular proteins with slightly lower similarity to approved drug targets. All 4479 druggable genes were collectively used for subsequent analysis.

### 2.2. pQTL Dataset

Cis-protein quantitative trait loci (cis-pQTLs) specifically refer to pQTLs located in close genomic proximity (cis position) to their target genes or proteins [17]. We used two sources of pQTL in this study. To screen the druggable viable proteins for subsequent analysis, we used the cis-pQTL data from the study conducted by Zheng et al., including 738 cis-SNPs of 734 proteins (Table S2) [18]. Then, we obtained pQTL data of the screened proteins from 4674 proteins in the deCODE database. This large-scale database integrates the plasma proteome with genetic factors and diseases [19]. These data were used as the main pQTL to identify potential drug targets for AD. The instrumental variables for cis-pQTL were selected using the following criteria: (1) *p*-value < 5 × 10^−8^; (2) SNPs in the human major histocompatibility complex (MHC) region were excluded [20]; (3) SNPs of 1 Mb upstream or downstream of the gene [21]; and (4) removal of linkage disequilibrium r^2^ < 0.1. The selected datasets were all of European ethnic background.

### 2.3. Outcomes Dataset

FinnGen is a large-scale research project focused on genomics and personalized medicine. It collects and analyzes genomic and health data from 500,000 Finnish biobank donors to gain a deeper insight into the genetic foundations of diseases. FinnGen R9 is the ninth data release of the FinnGen project. Our database from Finland R9 version provided https://storage.googleapis.com/finngen-public-data-r9/summary_stats/finngen_R9_I9_AORTDIS.gz (accessed on 2 April 2024) artery GWAS statistics for dissection. In addition, standardized summary association statistics obtained using the R package TwoSampleMR were used as outcomes in secondary analyses. In total, 881 patients with AD and 349,539 controls were included, and the outcome data were from European ethnic backgrounds.

### 2.4. Two-Sample MR

We performed a two-sample MR analysis using the TwoSampleMR package, with the druggable protein studied by Zheng et al. as the exposure factor and AD as the outcome [18].

We used the Wald ratio method to assess the results of MR for exposures containing only one SNP. Furthermore, we used the inverse variance weighted (IVW) method to assess the results of MR for exposures containing two or more SNPs. We used the TwoSampleMR package to conduct heterogeneity tests, pleiotropy tests, and leave-one-out analyses; assess the consistency of instrumental variables; eliminate the effects of multiple pathways; and ensure the robustness of the results. Then, we used the Steiger direction test to assess directionality and determine the correctness of the direction of causality.

Thereafter, we selected the proteins with a significant causal relationship with AD, downloaded the pQTL data of the corresponding proteins from the deCODE database as exposure factors, and performed a two-sample MR analysis considering AD as the outcome. Using the same approach, we used the Wald ratio to evaluate the MR effect for exposures containing only one SNP and the IVW method to evaluate the MR effect for exposures containing two or more SNPs. We used the TwoSampleMR package to conduct the heterogeneity test, pleiotropy test, and leave-one-out analysis. Then, we used the Steiger direction test to assess directionality and determine the correctness of the direction of causality.

### 2.5. SMR Analysis

Summary data-based MR (SMR) [4] uses GWAS summary data from GWAS and expression quantitative trait loci (QTL) studies to assess pleiotropic associations between base protein expression levels and complex traits of interest [22]. The heterogeneity in dependent instruments (HEIDI) test is used to assess the presence of possible horizontal pleiotropy in colocalization signals. The null hypothesis of the HEIDI test suggests no horizontal pleiotropy, which refers to a situation where a single genetic variant affects multiple traits in a pathway independent of the trait being analyzed [23]. The SMR and HEIDI methods can determine whether the effect of genetic variants (SNPs) on phenotypes occurs through protein expression or via alternative biological pathways. We downloaded the SMR Linux version (1.3.1) from the official SMR website https://yanglab.westlake.edu.cn/software/smr (accessed on 3 April 2024) and used the default parameters to perform SMR analysis.

### 2.6. Colocalization Analysis

We used the coloc package for colocalization analysis. The coloc package uses a Bayesian approach to assess the support for the following five exclusivity hypotheses: (1) SNPs are not associated with trait 1 or trait 2; (2) SNPs are associated with trait 1; (3) SNPs are associated with trait 2; (4) SNPs are associated with both trait 1 and trait 2, but they were independent SNPs; (5) SNPs were common to trait 1 and trait 2. The posterior probabilities of each test were H0, H1, H2, H3, and H4. To estimate the posterior probability of shared variation, for each selected protein, all SNPs within 250 kb upstream and downstream of its top SNP were retrieved for colocalization analysis. We considered PH4 > 0.8 as evidence of colocalization between GWAS and pQTL.

### 2.7. Drug Target Analysis

The protein–protein interaction (PPI) network is composed of individual proteins interacting with each other. The STRING database is used to search the interactions between known and predicted proteins [24]. In this study, we used the STRING database, set the biological species as human, and obtained the proteins that interacted with druggable proteins with a minimum correlation coefficient greater than 0.900 as the standard. We constructed the PPI network and used the R packages igraph and ggraph to visualize the PPI network model.

The DRUGBANK database includes FDA-approved drugs, drugs in clinical trials, and experimental drugs, providing detailed information on chemical structures, pharmacological properties, mechanisms of action, and drug interactions [25]. We searched the DRUGBANK database to obtain the drugs corresponding to all proteins included in PPI and their modes of action. The selected drugs could be used for treating AD in the later stage [25].

### 2.8. Quantitative Real-Time PCR (qPCR) Analysis

We verified the mRNA expression levels of the proteins identified through the PPI analysis in human aortic tissues through qPCR analysis. The human aortic tissues were obtained from AD patients (AD) undergoing aortic replacement surgery and from surplus aortic tissues excised from heart transplant donors (CONTROL).

RNA was extracted from human aortic tissues using TRIzol reagent (Cat. 15596026, Invitrogen, Waltham, MA, USA) according to the manufacturer’s instructions. The concentration and purity of the RNA were determined with a NanoDrop 2000 spectrophotometer (Cat. ND-2000, Thermo Scientific, Waltham, MA, USA), with A260/A280 ratios between 1.8 and 2.0. For cDNA synthesis, 1 µg of total RNA was reverse transcribed using HiScript III RT SuperMix (Cat. R323-01, Vazyme, Nanjing, China). The qPCR analysis was performed with a CFX96 Real-Time PCR Detection System (Bio-Rad, Hercules, CA, USA) in a 10 µL reaction volume containing 5 µL SsoFast EvaGreen Green Super Mix 500 RNX (Cat. 1725201, Bio-rad), 1 µL cDNA template, and 0.5 µM forward and reverse primers. Primer sequences are provided in Table S4. The results were analyzed using Bio-Rad CFX Maestro. Each sample was analyzed in triplicate technical replicates, and the experiment was repeated three times. The data are presented as mean ± standard deviation (SD). Statistical analysis was performed using Student’s *t*-test, with *p* < 0.05 considered statistically significant.

### 2.9. Statistical Methods

Data calculations and statistical analyses were performed using R software (https://www.r-project.org/, version 4.2.2). The data of qPCR analysis are presented as mean ± s.d. Comparison of two groups was performed using an unpaired *t*-test. Statistical analyses were performed using GraphPad Prism 9.0 (GraphPad Software Inc., San Diego, CA, USA). *p* (*p*-values) < 0.05 was considered statistically significant.

## 3. Results

### 3.1. Technology Roadmap

**Figure 1:**
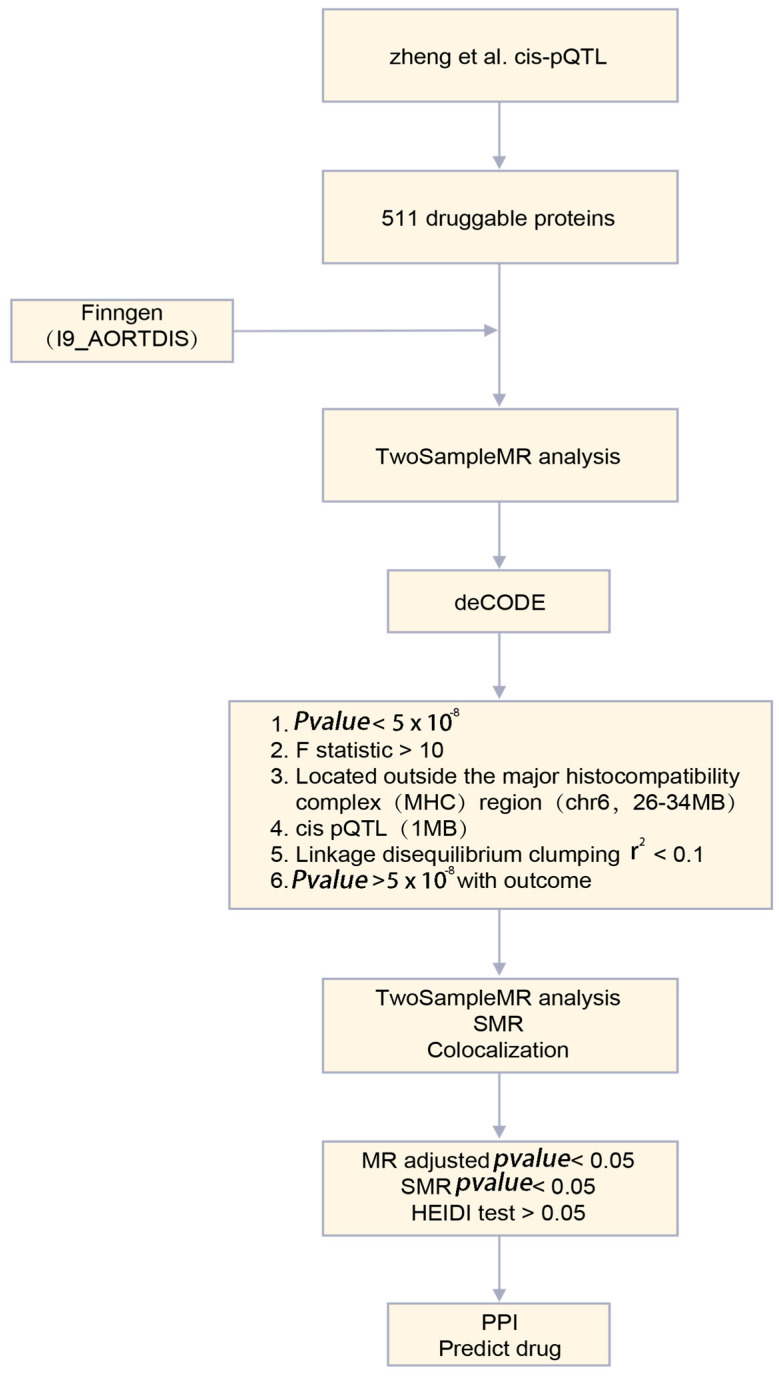
Technology Roadmap. MR, Mendelian randomization; SMR, summary-data-based Mendelian randomization; HEIDI, heterogeneity in dependent instruments; PPI, protein–protein interaction; pQTL, protein quantitative trait locus [18].

The analysis flow of this study is shown in Figure 1.

### 3.2. MR Analysis of Druggable Available Proteins

We first conducted an intersection analysis of 734 proteins studied by Zheng et al. [18]. using a dataset comprising 4479 proteins that are targets of existing patented drugs. This analysis yielded 511 proteins that were studied by Zheng et al. [18]. and served as targets for patented drugs, indicating their potential therapeutic relevance. Subsequently, we employed the TwoSampleMR package to assess the potential causal relationships between these proteins and AD. In the initial screening, a *p*-value < 0.05 was used as the threshold for significant causal screening. In total, 30 proteins had a causal relationship with AD (Table 1).

**Figure 2:**
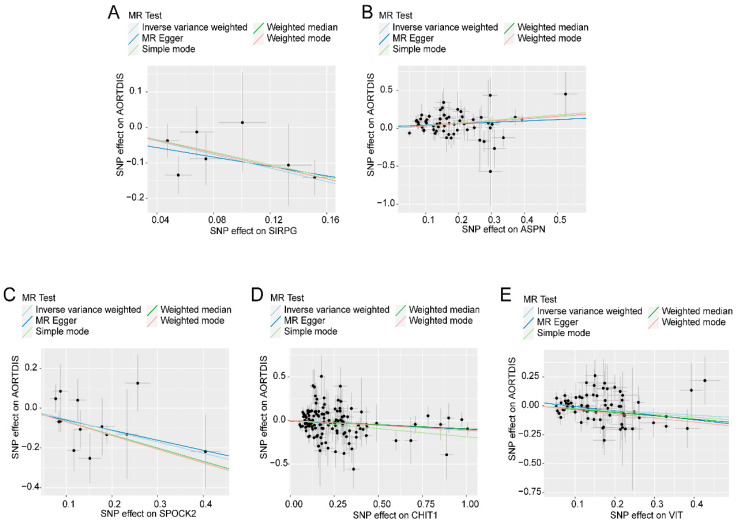
Scatter plot of effect estimates for different models of Mendelian randomization analysis of protein on aortic dissection. Scatter plot of different Mendelian randomization model effect estimates of (**A**) SRPG on aortic dissection; (**B**) scatter plot of different Mendelian randomization model effect estimates of ASPN on aortic dissection; (**C**) scatter plot of different Mendelian randomization model effect estimation of aortic dissection by ASPN; (**D**) scatter plot of different Mendelian randomization model effects of CHIT1 on aortic dissection; (**E**) scatter plot of Mendelian randomization different model effect estimates of VIT on aortic dissection.

Since each of the 30 proteins contained only one SNP, subsequent sensitivity analysis could not be performed. We downloaded the pQTL files of these 30 proteins from the deCODE database for subsequent analysis. First, we screened the pQTL files of the 30 proteins according to the screening criteria of cis-pQTL and obtained the corresponding cis-pQTL of the 30 proteins (see Table S3). Then, a two-sample MR analysis was performed on the 30 proteins and AD using TwoSampleMR. In the second screening, we used the more stringent Bonferroni test for *p*-value correction, and *p*.Adjust < 0.05 was used as the screening condition for significant causality to identify proteins with strong causal association with AD. In total, five proteins had a causal relationship with AD (Table 2), among which ASPN was positively correlated with the risk of AD, and SIRPG, SPOCK2, CHIT1, and VIT were negatively associated with the risk of AD. Finally, we drew a scatter plot of the effect estimates of different MR models for these five proteins and AD (Figure 2A–E). The intercept of each model line on the ordinate was close to 0, and the slope was in the same direction.

### 3.3. Sensitivity Analysis of Protein and AD

**Figure 3:**
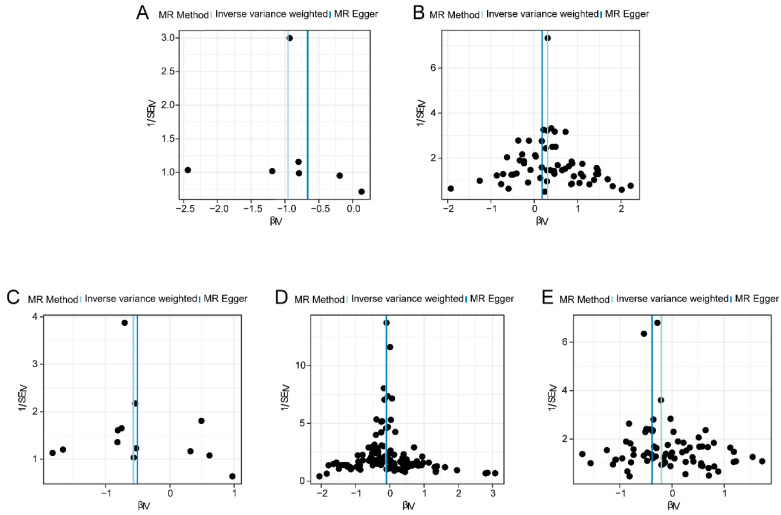
Funnel plot of heterogeneity test for Mendelian randomization analysis of protein on AD. (**A**) Funnel plot of Mendelian randomization for both protein and AD; (**B**) ASPN and AD Mendelian randomization funnel plot; (**C**) SPOCK2 and AD Mendelian randomization funnel plot; (**D**) Mendelian randomization funnel plot of CHIT1 and AD; (**E**) Mendelian randomization funnel plot of VIT and AD.

Firstly, we performed a heterogeneity test on five proteins (ASPN, SIRPG, SPOCK2, CHIT1, and VIT) and AD (Table 3). The results showed no heterogeneity in the MR results of four proteins (ASPN, SIRPG, SPOCK2, and VIT) for AD (I^2^ = 0, Cochran’s Q *p*-value > 0.05). There was slight heterogeneity in the MR results of CHIT1 for AD (Cochran Q *p*-value > 0.05, I^2^ < 25%). The funnel plot in Figure 3A–E shows that the instrumental variables of the five proteins were evenly distributed on the left and right sides of the IVW line, with no significant heterogeneity.

Then, we performed pleiotropy tests on the five proteins and AD (Table 4). The *p*-value of the pleiotropy tests for all proteins was greater than 0.05, and the intercept was close to 0, indicating that the causal inference was not affected by horizontal pleiotropy (Table 4).

Then, we conducted the leave-one-out test. All SNPs of the five proteins were measured using the leave-one-out method (Figure S1). The beta values of all results were closely aligned with 0, indicating negligible deviation from the null hypothesis. The high degree of overlap among the results suggests that the exclusion of any single SNP did not significantly alter the overall findings, and the results were robust.

We used the Steiger direction test to ensure that the causal direction of proteins affecting the pathogenesis of AD was correct. The *p*-value of the Steiger direction test of two proteins and AD was far less than 0.05, indicating the correct direction (Table 5).

### 3.4. SMR Analysis and Colocalization Analysis

The HEIDI test was used to confirm the existence of pleiotropy. We found that the *p*-value of the HEIDI test (p_HEIDI) for ASPN and SPOCK2 was > 0.05 (Table 6). This finding suggests no pleiotropy for the SNPs of these two proteins. The *p*-value of the SMR analysis was less than 0.05, proving a causal relationship between the two proteins and AD.

Based on the results of coloc analysis (see Table 7 for details), no protein had a high colocalization relationship with AD (PP.H4 > 0.8), and no protein had a moderate colocalization relationship with AD (0.5 < PP.H4 < 0.8).

### 3.5. Drug Target Analysis

**Figure 4:**
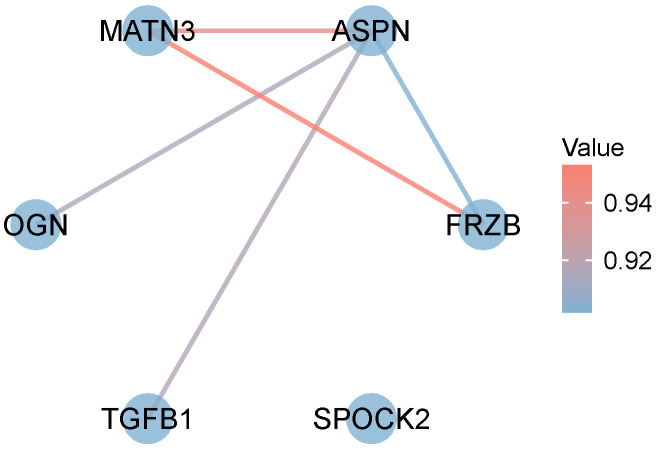
Analysis of PPI interaction network. PPI network, protein–protein interaction network. Nodes in the figure represent targets, and the color of the line from blue to red indicates the degree of correlation between nodes from small to large.

We extended the PPI analysis of two druggable targets (ASPN and SPOCK2) using the STRING database and constructed a set of six druggable-related targets (ASPN, FRZB, MATN3, OGN, TGFB1, MATN3, and SPOCK2) by retaining the targets with links to other nodes in the PPI network (Figure 4).

We analyzed the potential drugs of the target proteins in the DURGBANK database using six druggable-related targets. ASPN, FRZB, MATN3, OGN, and SPOCK2 had no available corresponding drugs (Table 8), indicating that these proteins are potential targets for drug discovery. Furthermore, proteins encoded by TGFB1 were targeted by six drugs (DB00070, DB06205, DB10770, DB10772, DB01162, and DB14740), suggesting that existing therapeutic interventions may affect AD through TGFB1.

**Figure 5:**
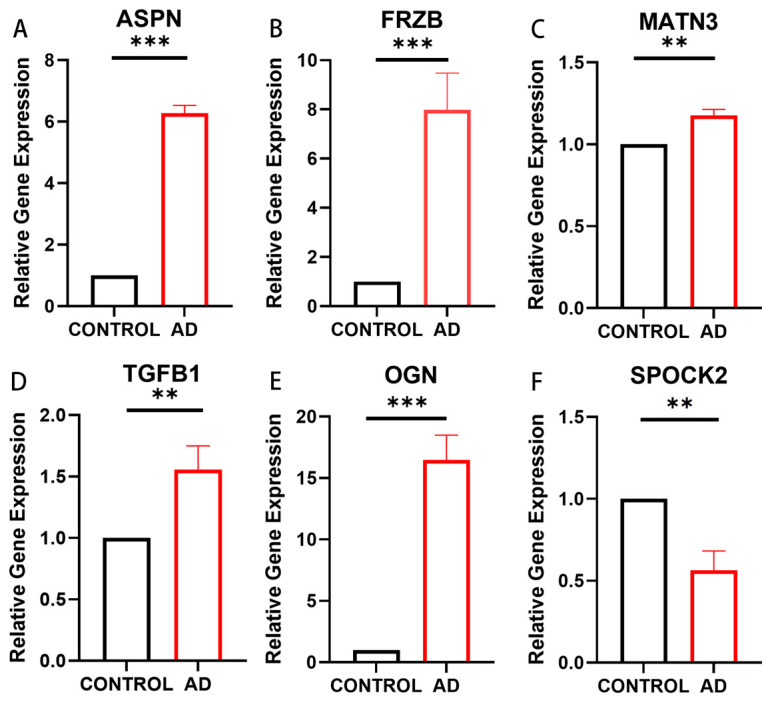
The mRNA expression of patent drug-related proteins in qPCR analysis (** indicates *p* < 0.001, *** indicates *p* < 0.0001). (**A**) The mRNA expression level of ASPN in the CONTROL group is lower than in the AD group (*p* < 0.0001). (**B**) The mRNA expression level of FRZB in the CONTROL group is lower than in the AD group (*p* < 0.0001). (**C**) The mRNA expression level of MATN3 in the CONTROL group is lower than in the AD group (*p* < 0.001). (**D**) The mRNA expression level of TGFB1 in the CONTROL group is lower than in the AD group (*p* < 0.001). (**E**) The mRNA expression level of OGN in the CONTROL group is lower than in the AD group (*p* < 0.0001). (**F**) The mRNA expression level of SPOCK2 in the AD group is lower than in the CONTROL group (*p* < 0.001).

We conducted qPCR analysis on the six patent drug-related proteins identified through PPI analysis to validate the mRNA expression levels of the corresponding genes. The results revealed that the expression levels of ASPN, FRZB, MATN3, OGN, and TGFB1 were significantly lower in the CONTROL group compared to the AD group, with statistically significant differences. Conversely, the expression level of SPOCK2 was significantly higher in the CONTROL group than in the AD group, also with statistically significant differences. These findings are consistent with our MR results, indicating that the mRNA expressions of ASPN, FRZB, MATN3, OGN, and TGFB1 are positively correlated with AD, while the mRNA expression of SPOCK2 is negatively correlated with AD. The qPCR experimental results not only corroborated the protein validation MR but also provided important insights into the molecular mechanisms of AD (Figure 5). These discoveries lay a solid foundation for the subsequent exploration of therapeutic targets for AD.

## 4. Discussion

This study utilized drug-targeted MR analysis, employing genetic variants as instrumental variables free from confounding factors, to explore the relationship between druggable proteins and the risk of AD. We aimed to identify potential therapeutic targets by understanding the genetic basis of diseases. To elucidate the correlation between genetic mutations and gene expression in AD, we intersected 734 proteins and 4479 genes identified as druggable. We identified 511 genes encoding proteins with potential therapeutic relevance [18]. MR analysis focused on proteins encoded in the context of AD. Five proteins, including ASPN, SIRPG, SPOCK2, CHIT1, and VIT, were identified in secondary screening. These proteins demonstrated strong causal associations with the risk of AD. Our findings reveal that ASPN exhibits a positive association with the risk of AD, suggesting an increased risk with higher ASPN levels. Conversely, SIRPG, SPOCK2, CHIT1, and VIT showed negative associations, indicating these proteins might confer protective effects against AD.

Using SMR, we verified the horizontal pleiotropy of the proteins and found no pleiotropy in the SNPs of the ASPN and SPOCK proteins. PPI analysis was expanded for the two druggable targets (ASPN and SPOCK2) using the STRING database after retaining the targets with links to other nodes. Finally, the TGFB1-encoded protein was found to correspond to six drugs in DRUGBANK, including DB00070, DB06205, DB10770, DB10772, DB01162, and DB14740, suggesting that existing therapeutic interventions could potentially affect the pathology of AD through the biological pathways of TGFB1. We found no corresponding drugs for the proteins encoded by ASPN, FRZB, MATN3, OGN, and SPOCK2 (Table 8). We conducted qPCR analysis on the mRNA expression levels of the six identified patent druggable proteins (Figure 5). The results at the mRNA level validated the protein findings, which are consistent with the previous MR results: SPOCK2 (OR 0.54 (0.35, 0.84)) and ASPN (OR 1.29 (1.03, 1.61)). These results indicate that inhibiting ASPN, FRZB, MATN3, OGN, and TGFB1 expression may aid in treating AD, while activating SPOCK2 expression could prevent AD. This provides important insights into the molecular mechanisms of AD and lays a foundation for exploring therapeutic targets for AD.

MR represents a powerful tool for enhancing the efficacy of current cardiovascular medications and identifying novel therapeutic agents. By elucidating the relationships between specific genetic variants and AD, MR facilitates the identification of novel drug targets. It unravels the relationship between specific genetic variants and AD, which enables the identification of new drug targets. This, in turn, facilitates the design and development of drugs that can act on these targets [26].

While this study successfully identified proteins significantly associated with AD, it did not yield existing drugs capable of directly targeting the five proteins with strong causal links to AD. This highlights a gap in the current therapeutic options that necessitates further exploration. In subsequent PPI analyses, we identified six drugs targeting proteins encoded by TGFB1, suggesting potential indirect pathways for therapeutic intervention in AD. Given the intricate etiology of AD, characterized by multifaceted genetic factors and the potential interplay between various signaling pathways, our findings underscore the importance of using existing drugs. This step is crucial to confirm the therapeutic relevance of the identified targets and drugs.

TGFB1 is a multifunctional cytokine belonging to the TGF-β superfamily, and members of this superfamily play key roles in many biological processes, including cell proliferation, differentiation, apoptosis, and other cellular functions [27,28].

TGF-β plays a central role in inducing fibrosis [29]. It promotes fibroblast activation and differentiation and increases the production of collagen and other components of the extracellular matrix [30]. Mutations and defects of TGF-β1 and fibrillin1 (FBN1) increase arterial injury response [31]. In addition, the TGF-β signaling pathway is a potential upstream driver of SMC [32]. The above studies illustrate the potential relationship between TGF-β1 and AD. Previous studies have demonstrated that TGF-β1 inhibits inflammation by promoting macrophage-mediated phagocytosis [33]. TGF-β1 also activates the pro-angiogenic Smad1/5/8 signaling pathway [34]. On the other hand, TGF-β1 is involved in the pathogenesis of many cardiovascular diseases. Increased activity of the TGF-β signaling pathway contributes to structural vulnerabilities of the connective tissues. Activation of TGF-β and its downstream pathways plays a pivotal role in the complex pathogenesis of aortic aneurysm and dissection, highlighting its significance in these conditions [35,36].

The expression of TGF-β1 and related core genes, such as the Smad family, is closely associated with aortic aneurysms and aortic clamps, suggesting that targeted therapy with TGF-β1 provides a theoretical foundation [37,38]. Previous studies have demonstrated that angiotensin II receptor blockers, particularly losartan, can inhibit TGF-β expression. This finding suggests a potential therapeutic avenue for mitigating the adverse effects of the TGF-β signaling pathway in conditions like aortic aneurysm and dissection [39]. Both in vitro and in vivo experiments demonstrated that inhibition of TGF-β1 maturation, alongside inhibiting the phosphorylation of classical signaling pathways, such as Smad2/3, the mitogen-activated protein kinase (MAPK) signaling pathway, and intracellular signal-regulated kinases 1 and 2 (ERK1/2), in vascular smooth muscle cells (VSMCs) can significantly inhibit the development of thoracic aortic aneurysm [40,41]. In addition, the TGF-β1 signaling pathway is associated with the death receptor apoptotic pathway (Fas and TNF), intracellular apoptosis regulator c-Jun N-terminal kinase (JNK), and phosphoinositide 3-kinase/Akt (PI3K/Akt pathway) [42,43].

Higher-level evidence is needed for the application of TGF-β1 as a drug target. Despite these promising findings in vitro, more studies are needed to assess the therapeutic efficacy of TGF-β-targeting drugs in treating AD.

This underscores the importance of ongoing studies for exploring and validating potential treatments. MR can help eliminate the bias often found in traditional clinical studies. This method provides a reliable and evidence-based rationale for potential drug targets that have been established in animals and cells but need validation through rigorous clinical trials [44]. MR allows for researchers to pinpoint drug targets with a genetically supported likelihood of influencing the disease process, thereby advancing the development of drug candidates with a greater potential for clinical use [45].

Using MR, we illustrated that the interaction between TGFB1 and ASPN leads to the potential preventive or therapeutic effects of TGF-β inhibitors on AD.

This study established a causal link between specific genetically determined druggable proteins and the risk of AD through MR. Furthermore, our study pinpointed several proteins with a strong association with AD, underscoring their potential as targets for therapeutic intervention. For individuals at high risk of developing AD, including those with pre-existing aortic aneurysms or hypertension, our findings hold significant promise for future personalized treatment. AD is a life-threatening acute aortic syndrome for which surgical intervention is the primary treatment. Our findings may be useful for patients undergoing conservative or complementary treatment. Especially in chronic AD, TGF-β inhibitors can ameliorate vascular damage and VSMC dysfunction [31,46]. This may be beneficial for precision medicine in this population [47].

Additionally, these insights could be instrumental in shaping the design of future clinical study protocols, potentially revolutionizing how we approach this complex condition [48].

Due to the lack of outcome GWAS data for AD, it was impossible to analyze different types of the disease. For example, in terms of drug targets, there may be some differences between patients with AD according to the Stanford classification system [49]. It is important to acknowledge the limited diversity of sources for druggable proteins and associated SNP data. Further studies are needed to ascertain if identified druggable proteins and potential pharmacological agents exert consistent therapeutic effects across different subtypes of AD. GWAS data sources utilized for analyzing disease outcomes represent genetic variations primarily from populations of European descent, which limits their applicability across other ethnic groups. To enhance the external validity of our findings, future research efforts must consider including populations from different regions and ethnicities. In addition, the biological pathways through which the druggable proteins affect AD are not fully understood. Investigating epigenetic regulation to understand the interplay between genetic susceptibility and environmental exposures in the development of AD is crucial for establishing future preventive or therapeutic strategies [50,51].

## 5. Conclusions

Using MR analysis, we found significant causal relationships between six druggable targets (ASPN, FRZB, MATN3, OGN, TGFB1, MATN3, and SPOCK2) and AD, suggesting that these genes can serve as potential therapeutic targets. A focused analysis of ASPN and SPOCK2 revealed their potential effect on AD as risk factors. Additionally, through drug-targeting analysis, we identified six drugs that can interact with the TGFB1 protein encoded by the ASPN gene. These interactions offer promising avenues for the development of targeted therapeutics. Future studies should fill the gap between MR and real-world clinical trials. For example, in vivo and in vitro studies are needed to validate the results [52].

## Figures and Tables

**Table 1:** Mendelian randomization causal effect estimates of the druggable proteins on the onset of aortic dissection from the study by Zheng et al. [18].

Exposure	Outcome	nsnp	b	se	OR (95% CI)	*p* Value	Method
THSD1	AORTDIS	1	0.488148	0.226678	1.63 (1.04, 2.54)	0.031281	Wald ratio
MCAM	AORTDIS	1	0.85871	0.376192	0.42 (0.20, 0.89)	0.022452	Wald ratio
NOV	AORTDIS	1	0.70118	0.304777	0.50 (0.27, 0.90)	0.021413	Wald ratio
CP	AORTDIS	1	0.48638	0.215355	0.61 (0.40, 0.94)	0.023914	Wald ratio
FCGR3B	AORTDIS	1	0.35885	0.127644	0.70 (0.54, 0.90)	0.004933	Wald ratio
CST7	AORTDIS	1	0.24508	0.10313	0.78 (0.64, 0.96)	0.017482	Wald ratio
TGFB1	AORTDIS	1	0.761629	0.204704	2.14 (1.43, 3.20)	0.000199	Wald ratio
APOH	AORTDIS	1	0.43335	0.147132	0.65 (0.49, 0.87)	0.003226	Wald ratio
APOB	AORTDIS	1	0.399538	0.159468	1.49 (1.09, 2.04)	0.012229	Wald ratio
ICAM1	AORTDIS	1	0.10545	0.03988	1.11 (1.03, 1.20)	0.008189	Wald ratio
FABP1	AORTDIS	1	0.51988	0.252266	0.59 (0.36, 0.97)	0.039319	Wald ratio
ADH4	AORTDIS	1	0.7258	0.30987	0.48 (0.26, 0.89)	0.019166	Wald ratio
ADH5	AORTDIS	1	0.538683	0.191132	1.71 (1.18, 2.49)	0.004827	Wald ratio
PTHLH	AORTDIS	1	0.718727	0.302689	2.05 (1.13, 3.71)	0.017574	Wald ratio
CTRB1	AORTDIS	1	0.22457	0.092959	0.80 (0.67, 0.96)	0.015699	Wald ratio
AZU1	AORTDIS	1	0.45577	0.23165	0.63 (0.40, 1.00)	0.049127	Wald ratio
GPC5	AORTDIS	1	0.30833	0.146734	0.73 (0.55, 0.98)	0.035614	Wald ratio
CHIT1	AORTDIS	1	0.13044	0.056318	0.88 (0.79, 0.98)	0.020547	Wald ratio
FCRL6	AORTDIS	1	0.294386	0.114034	1.34 (1.07, 1.68)	0.009835	Wald ratio
SPINK6	AORTDIS	1	0.198157	0.075838	1.22 (1.05, 1.41)	0.008978	Wald ratio
VIT	AORTDIS	1	0.51051	0.149513	0.60 (0.45, 0.80)	0.000639	Wald ratio
CHRDL2	AORTDIS	1	0.44762	0.182955	0.64 (0.45, 0.91)	0.01442	Wald ratio
SPOCK2	AORTDIS	1	0.61669	0.223173	0.54 (0.35, 0.84)	0.005722	Wald ratio
GGH	AORTDIS	1	0.23639	0.102127	0.79 (0.65, 0.96)	0.020631	Wald ratio
SPON2	AORTDIS	1	0.575535	0.238549	1.78 (1.11, 2.84)	0.015837	Wald ratio
ASPN	AORTDIS	1	0.255	0.113223	1.29 (1.03, 1.61)	0.02431	Wald ratio
CBLN4	AORTDIS	1	0.57246	0.290034	0.56 (0.32, 1.00)	0.048409	Wald ratio
SIRPG	AORTDIS	1	0.5362	0.19223	0.58 (0.40, 0.85)	0.005281	Wald ratio
DKK2	AORTDIS	1	0.307139	0.135913	1.36 (1.04, 1.77)	0.023833	Wald ratio
SIGLEC9	AORTDIS	1	0.09608	0.03997	0.91 (0.84, 0.98)	0.01623	Wald ratio

SNP, single-nucleotide polymorphism; OR, odds ratio; CI, confidence interval.

**Table 2:** Mendelian randomization causal effect estimates of druggable proteins on the onset of aortic dissection from deCODE.

Exposure	Outcome	nsnp	b	se	OR (95% CI)	*p* Value	*p*.Adjust	Method
SIRPG	AORTDIS	7	0.95509	0.259728	0.38 (0.23, 0.64)	2.36 × 10^−4^	5.89 × 10^−3^	Inverse variance weighted
ASPN	AORTDIS	61	0.308425	0.064425	1.36 (1.20, 1.54)	1.69 × 10^−6^	4.22 × 10^−5^	Inverse variance weighted
SPOCK2	AORTDIS	13	0.56615	0.161402	0.57 (0.41, 0.78)	4.52 × 10^−4^	1.13 × 10^−2^	Inverse variance weighted
CHIT1	AORTDIS	128	0.1156	0.033833	0.89 (0.83, 0.95)	6.34 × 10^−4^	1.59 × 10^−2^	Inverse variance weighted
VIT	AORTDIS	73	0.20247	0.061502	0.82 (0.72, 0.92)	9.95 × 10^−4^	2.49 × 10^−2^	Inverse variance weighted

SNP, single-nucleotide polymorphism; OR, odds ratio; CI, confidence interval.

**Table 3:** Heterogeneity test of Mendelian randomization analysis of proteins on AD.

Exposure	Outcome	Q	Q_df	Q_pval	I^2^ (%)
SIRPG	AORTDIS	3.609128058	6	0.729398604	0
ASPN	AORTDIS	57.98267787	60	0.54981067	0
SPOCK2	AORTDIS	11.72220555	12	0.468239735	0
CHIT1	AORTDIS	159.8957593	127	0.025604157	20.57
VIT	AORTDIS	73.41351092	72	0.431509416	1.93

Q, Cochran Q test statistic; Q_df, Q test degree of freedom; Q_pval, *p* value of Q test; the I^2^ statistic reflects the proportion of the heterogeneity part of the instrumental variable in the total variation: if I^2^ ≤ 0, it is set to 0, indicating that no heterogeneity is observed. I^2^ = 0–25%, indicating mild heterogeneity; I^2^ = 25–50%, indicating moderate heterogeneity; I^2^ > 50% indicating high heterogeneity. The specific calculation formula is I^2^ = (q − df)/Q × 100%.

**Table 4:** Pleiotropy tests for Mendelian randomization analysis of protein to AD.

Exposure	Outcome	egger_intercept	se	pval
SIRPG	AORTDIS	0.03044	0.054953	0.603505
ASPN	AORTDIS	0.023685	0.021724	0.280028
SPOCK2	AORTDIS	0.0094	0.056081	0.869955
CHIT1	AORTDIS	0.00798	0.012463	0.523339
VIT	AORTDIS	0.032323	0.0183	0.081639

**Table 5:** Steiger directionality test for Mendelian randomization analysis of proteins for AD.

Exposure	Outcome	snp_r2.exposure	snp_r2.outcome	correct_causal_direction	steiger_pval
SIRPG	AORTDIS	0.015718	4.89 × 10^−5^	TRUE	5.09 × 10^−101^
ASPN	AORTDIS	0.232303	0.000231	TRUE	0
SPOCK2	AORTDIS	0.039031	6.86 × 10^−5^	TRUE	2.63 × 10^−259^
CHIT1	AORTDIS	0.600093	0.000498	TRUE	0
VIT	AORTDIS	0.221213	0.000241	TRUE	0

SNP, single-nucleotide polymorphism; r^2^, variance explained rate.

**Table 6:** Results of SMR analysis of proteins for aortic dissection.

Gene	Exposure	Outcome	topSNP	nsnp_HEIDI	p_SMR	p_HEIDI
ENSG00000133063.11	CHIT1	AORTDIS	rs2486959	6	1.86 × 10^−1^	6.74 × 10^−1^
ENSG00000205221.8	VIT	AORTDIS	rs12053542	4	5.63 × 10^−2^	1.52 × 10^−1^
ENSG00000106819.7	ASPN	AORTDIS	rs10992273	12	2.27 × 10^−2^	4.80 × 10^−1^
ENSG00000107742.8	SPOCK2	AORTDIS	rs1245548	4	6.82 × 10^−3^	6.65 × 10^−1^
ENSG00000089012.10	SIRPG	AORTDIS	rs149538314	NA	3.58 × 10^−1^	NA

SMR, summary-data-based Mendelian randomization; HEIDI, heterogeneity in dependent instruments; SNP, single-nucleotide polymorphism.

**Table 7:** Results of colocalization analysis of protein and AD coloc.

Exposure	Outcome	PP.H0.abf	PP.H1.abf	PP.H2.abf	PP.H3.abf	PP.H4.abf
ASPN	AORTDIS	0.00	0.6421004	0.00	0.177	0.181
SPOCK2	AORTDIS	6.52 × 10^−111^	0.4665417	2.36 × 10^−111^	0.169	0.365

**Table 8:** Drug information of patent drug-related targets in DRUGBANK.

Target	Uniprot	Drugbank ID	Name	Durg Group	Pharmacological Action	Actions
ASPN	Q9BXN1	NA	NA	NA	NA	NA
FRZB	Q92765	NA	NA	NA	NA	NA
MATN3	O15232	NA	NA	NA	NA	NA
OGN	P20774	NA	NA	NA	NA	NA
TGFB1	P01137	DB00070	Hyaluronidase (ovine)	approved, investigational	unknown	inhibitor
		DB06205	Hyaluronidase (human recombinant)	approved, investigational	unknown	inhibitor
		DB10770	Foreski fibroblast (neonatal)	approved	unknown	agonist
		DB10772	Foreskin keratinocyte (neonatal)	approved	yes	agonist
		DB01162	Terazosin	approved	yes	inducer
		DB14740	Hyaluronidase	approved	unknown	inhibitor
SPOCK2	Q92563	NA	NA	NA	NA	NA

## Data Availability

The original contributions presented in this study are included in the article/Supplementary Material; further inquiries can be directed to the corresponding author.

## Supplementary Materials

The following supporting information can be downloaded at: https://www.mdpi.com/article/10.3390/biomedicines12061204/s1, Figure S1: Consequence of leave-one-out analyses; Table S1: Locations and druggability priorities of all druggable genes.; Table S2: cis-pQTL data of 738 druggable proteins. Table S3: cis-pQTL data of 30 Proteins with causal relationships in aortic dissection; Table S4: Primer information used in qPCR.
