# Supplementary figures and images for: Uncovering Druggable Targets in Aortic Dissection: An Association Study Integrating Mendelian Randomization, pQTL, and Protein–Protein Interaction Network
Source: Biomedicines. 2024 May 29;12(6):1204. doi: 10.3390/biomedicines12061204 (PMC11200553; doi:10.3390/biomedicines12061204)

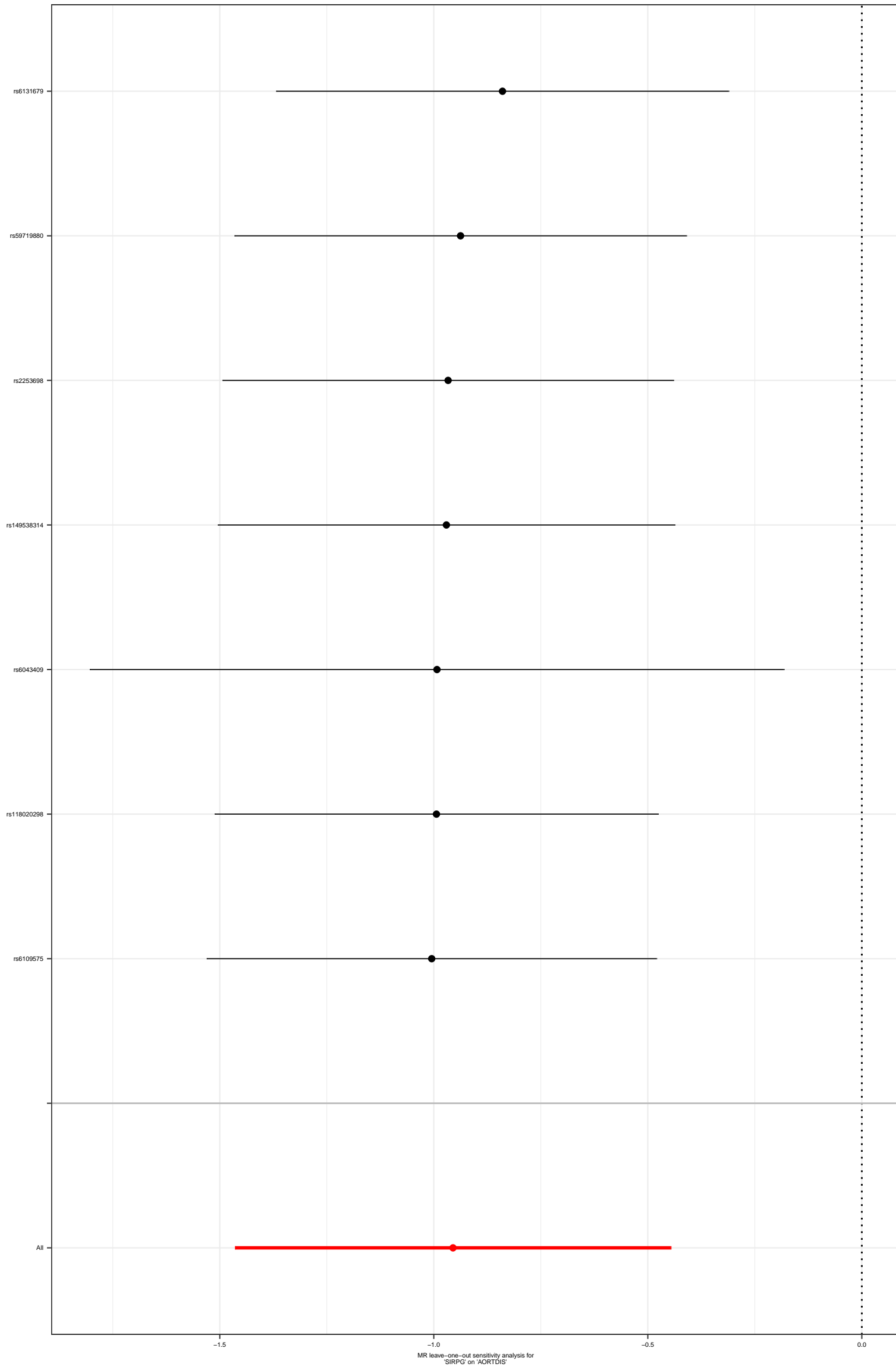

Supplement: Supplementary file 1 [file biomedicines-12-01204-s001.zip › SupplementaryFigure/mr_leaveoneout_plot-ENSG00000089012.10-xv7MQs.pdf]

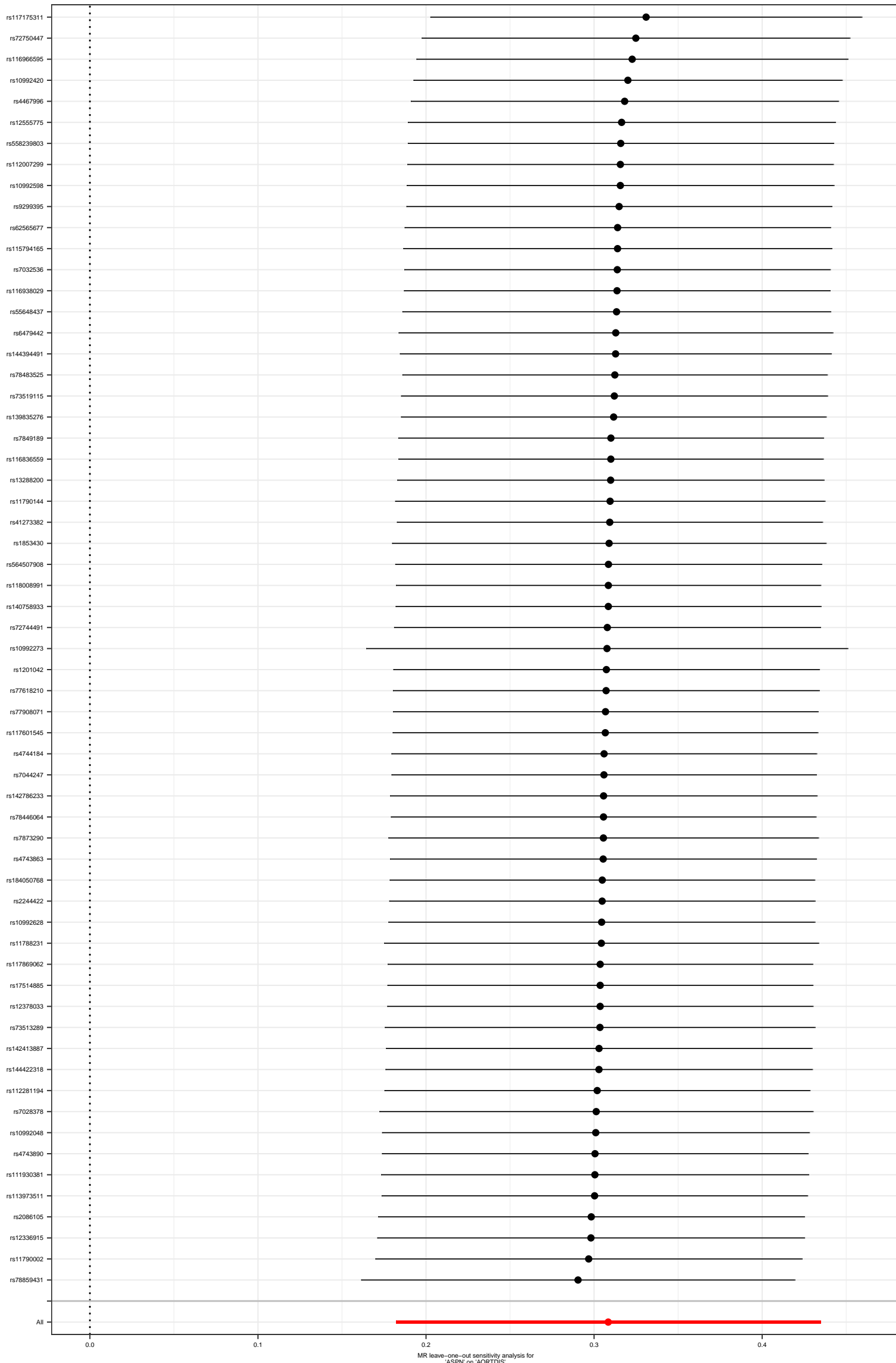

Supplement: Supplementary file 1 [file biomedicines-12-01204-s001.zip › SupplementaryFigure/mr_leaveoneout_plot-ENSG00000106819.7-xv7MQs.pdf]

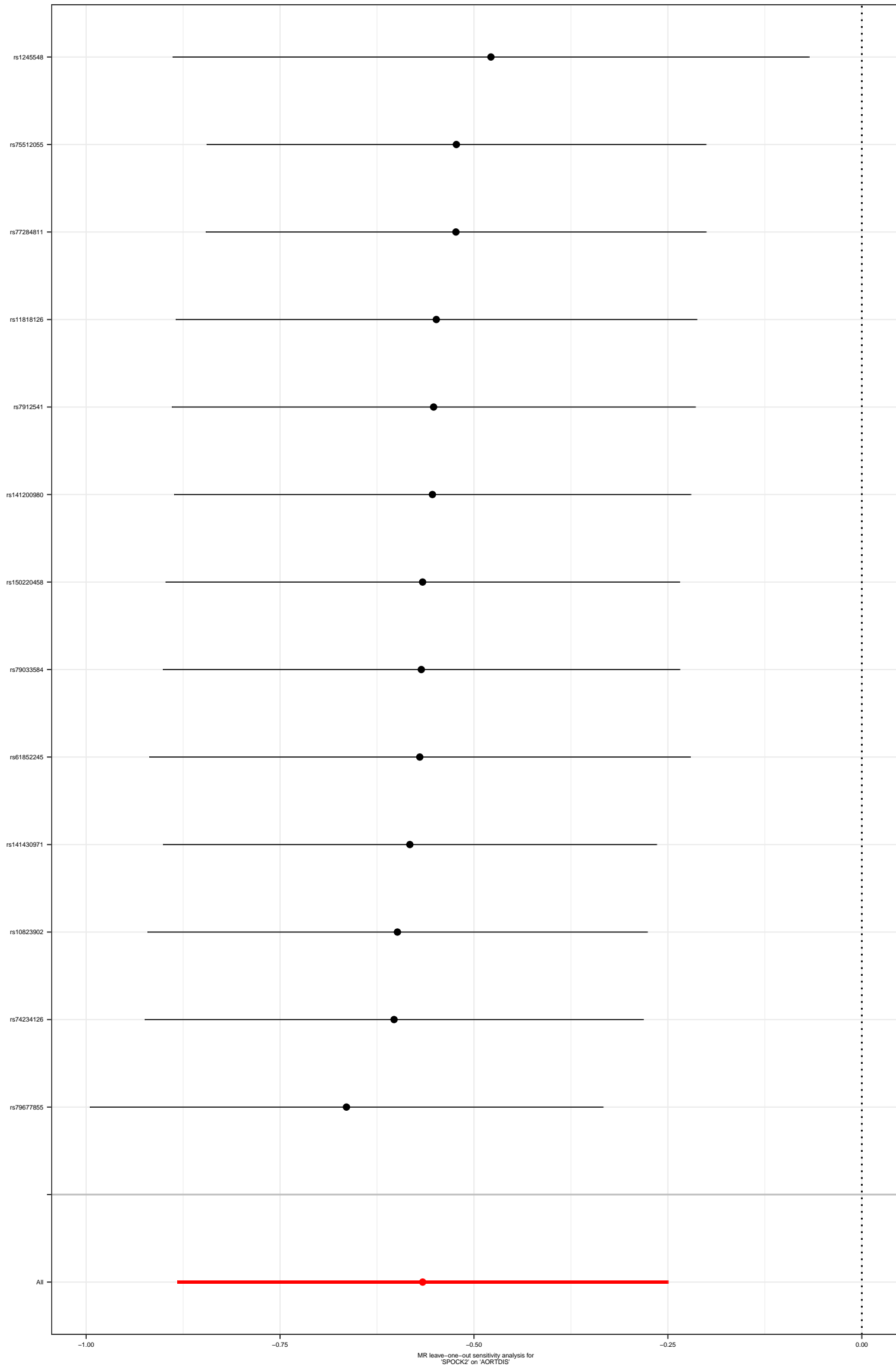

Supplement: Supplementary file 1 [file biomedicines-12-01204-s001.zip › SupplementaryFigure/mr_leaveoneout_plot-ENSG00000107742.8-xv7MQs.pdf]

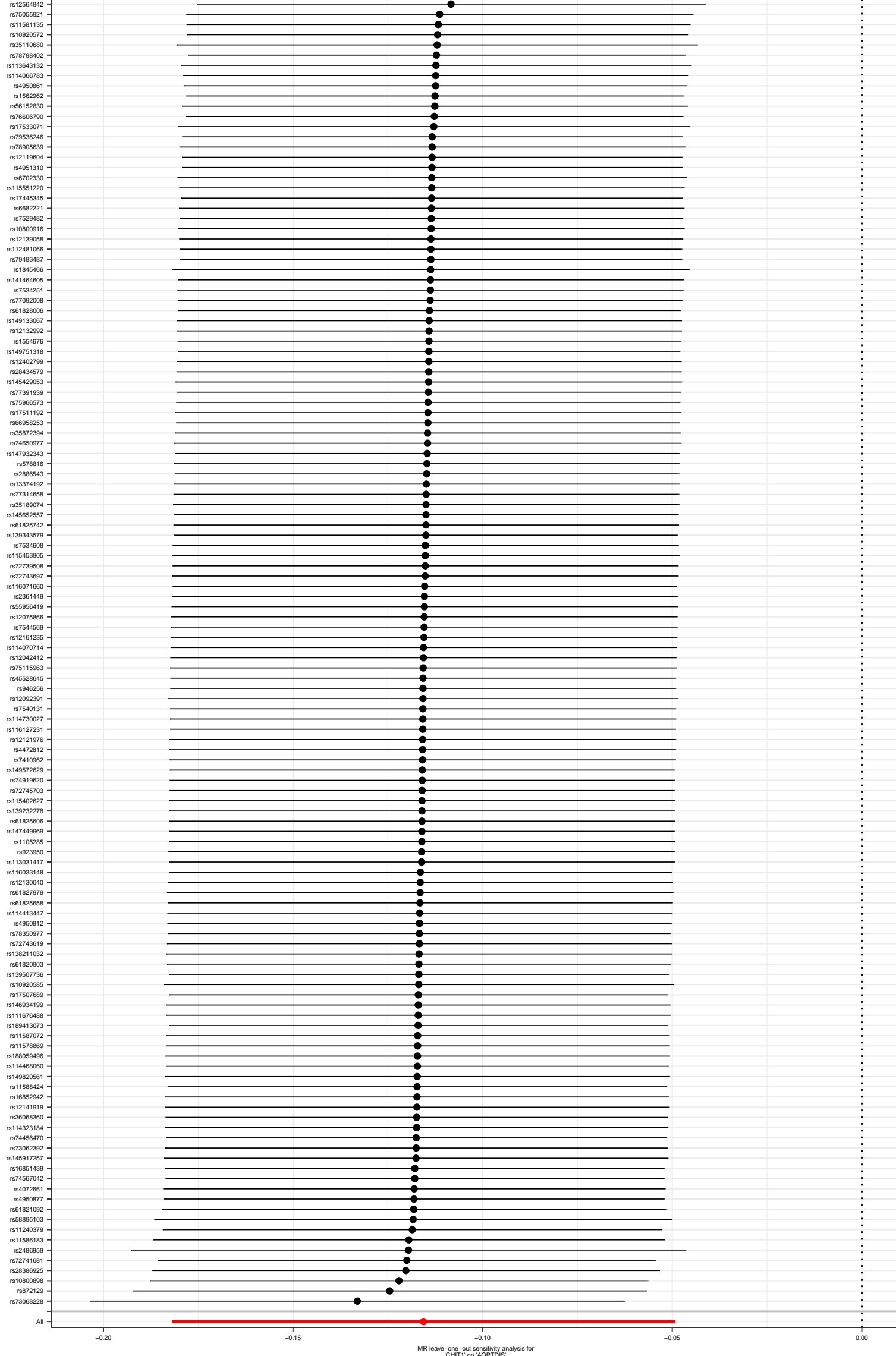

Supplement: Supplementary file 1 [file biomedicines-12-01204-s001.zip › SupplementaryFigure/mr_leaveoneout_plot-ENSG00000133063.11-xv7MQs.pdf]

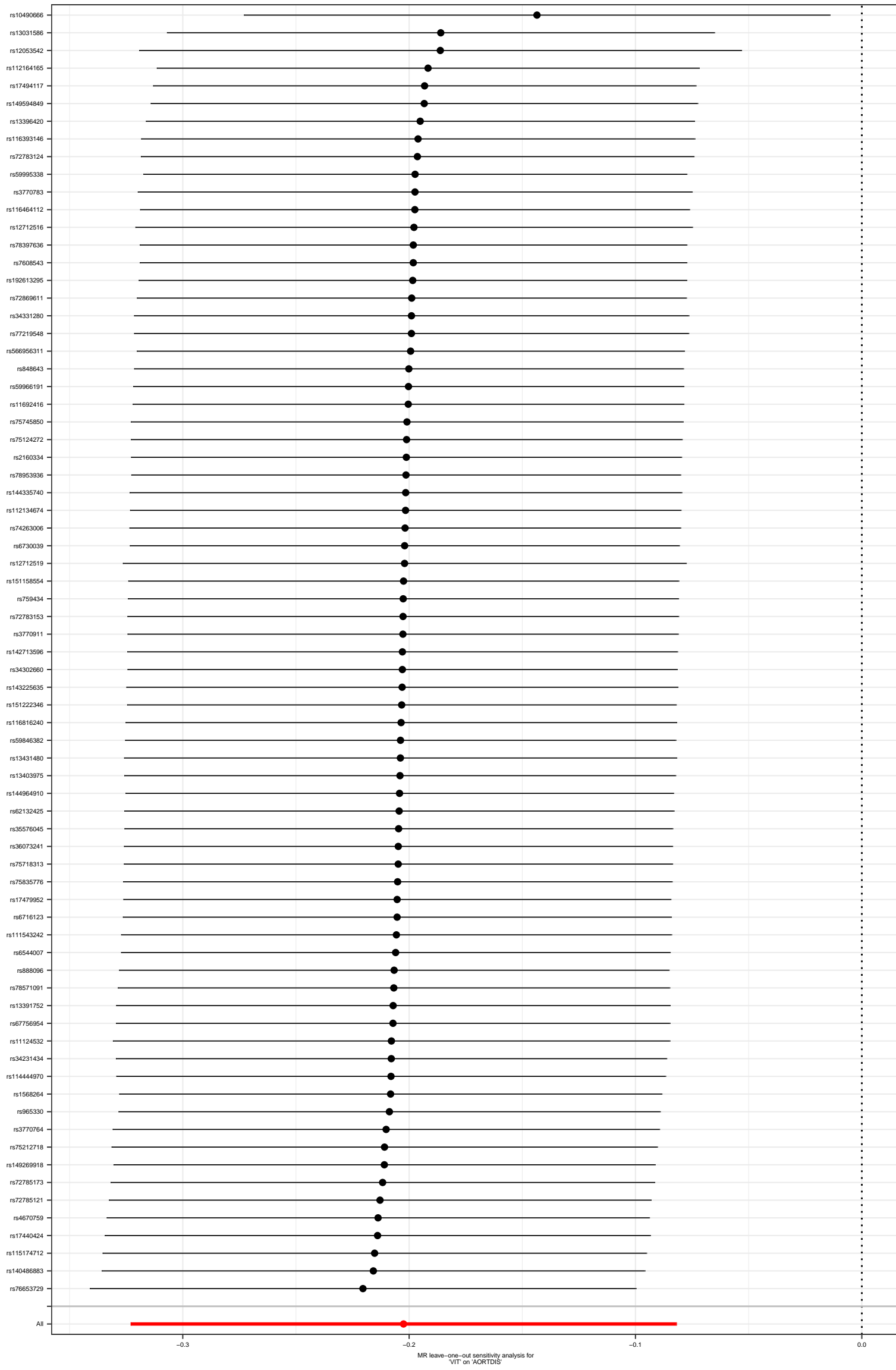

Supplement: Supplementary file 1 [file biomedicines-12-01204-s001.zip › SupplementaryFigure/mr_leaveoneout_plot-ENSG00000205221.8-xv7MQs.pdf]
